# Failure to comply with the law of freedom of right turn at intersections by drivers and its impact on the equation of Cost -benefit and the behavioral architecture of a Healthy City 

**Published:** 2019-08

**Authors:** Reza Hekmatshoar, Ahmad Allahabadi, Mohmmad Hasan Rakhshani, Fateme Abareshi, Taherh Azmon

**Affiliations:** ^*a*^ Department of Occupational Health and Safety Work Engineering, Collage of Health, Sabzevar University of Medical Sciences, Sabzevar, Iran.; ^*b*^ Department of Environment Health Engineering Collage of Health, Sabzevar University of Medical Sciences, Sabzevar, Iran.; ^*c*^ Department of Biostatistics, College of Health, Sabzevar University of Medical Sciences, Sabzevar, Iran.; ^*d*^ Master of Science in Persian Literature , sabzevar Education Directorate, Sabzevar, Iran.

## Abstract

**Background:**

The Present age , Known as the age of Nano science and fundamental particles in the world of science and technology, and developing countries are enchanted by the forms of modernity of the advanced world, is necessary to benefit from the Patterns of movement, including the ideas of humanity and the integration of the components of a community, the work it is a systematic, coordinated and beautiful way to institutionalize a culture of safety and health in the context of the goals of a healthy city that can be a platform for sustainable development. In this regard, it is necessary to look at the cultures of the ruling community and consider the impact of each of the indicators of Life style, including norms, values, beliefs, habits, traditions and customs .With the global thinking and regional thinking, it is possible to act smoothly through the hard way of architecture of a society and a healthy city, with the cost-benefit equation for increasing GDP and reaching a sustainable development. Therefore, failure to observe the right of right turn right at the intersections by drivers and its impact on the behavioral architecture of a healthy city as one of the forgotten indicators in sustainable development and its impact on community health in this research.

**Methods:**

The Present study was descriptive and analytical. The sample of 380 sample drivers required by systematic method during six months and between 17 and 20 nights in cooperation with Raw Police Personnel was used by drivers who had committed violations of right-turn right in two. The main intersection was collected and then analyzed by SPSS software version 16/5 using the Kolmogorov-Smirnov test, Normal filter and Chi – square.

**Results:**

Relationship was observed between .occupation type and attitude (P=0.013) ‚ occupational history and attitude (P=0.03) ‚ as well as residential area and attitude (P=0.011)‚ however‚ no significant relationship was found between age and attitude (P=0.0149)‚ education and attitude (P=0.0118) ‚ at a confidence interval of 95%. Total wasted time per year at a crossroad with three outlets was 1/123/260 hours per year. Given a 4 – cross road typical city in Iran with 340 cities ‚ an estimated 175/219 Dollar per year will be wasted. As far as the cost is concerned ‚ the total amount of gas wasted (10 Cent/liter) will amount to 175/219 Dollar per year in one single cross road in Sabzevar ‚Iran .The same equation applies here to produce a cost estimation of 240/000/000 Dollar /year nationwide.

**Conclusions:**

Immediate action for geometric correction of crossroads is required for preventing wastage of national resources and imposing economic costs on community members and the wasted time due to abnormal traffic behavior‚ increased GNP and moving toward Sustainable development will follow as a Consequence. Key words: Cost Benefit‚ GNP‚ Sustainable Development‚ Occupational Health Engineering.

**Keywords:**

Cost Benefit‚ GNP‚ Sustainable Development‚ Occupational Health Engineering

